# Supplementary material for: Impact of Socioeconomic Inequalities on Dental Caries Status in Sardinian Children
Source: Children (Basel). 2024 Jan 12;11(1):96. doi: 10.3390/children11010096 (PMC10814925; doi:10.3390/children11010096)
Supplement: Supplementary file 1 [file children-11-00096-s001.zip › children-2787264-supplementary.pdf]

Table S1. Inhabitants, 3-11 years old population (sorted by gender) of the municipalities involved in the study, with the related IDMS score.

Municipality	Inhabitants	3-11 years old	Females 3-11 years old	Males 3-11 years old	IDMS
Aggius	1468	87	47	40	0.32
Aglientu	1240	75	36	39	0.59
Alá dei Sardi	1837	167	90	77	0.68
Alghero	42760	2660	1291	1369	0.23
Anela	620	38	19	19	0.34
Ardara	766	53	26	27	0.79
Arzachena	13477	1102	549	553	0.21
Badesi	1850	120	53	67	0.55
Banari	528	31	20	11	0.58
Benetutti	1809	112	61	51	0.59
Berchidda	2689	169	72	97	0.57
Bessude	411	17	9	8	0.45
Bonnanaro	950	42	27	15	0.59
Bono	3481	264	140	124	0.2
Bonorva	3323	177	71	106	0.28
Bortigiadas	752	54	25	29	0.45
Borutta	279	13	6	7	0.92
Bottidda	673	45	13	32	0.42
Buddusò	3799	388	171	217	0.37
Budoni	5156	348	173	175	0.21
Bultei	897	43	20	23	0.36
Bulzi	492	23	8	15	0.55
Burgos	899	67	22	45	0.43
Calangianus	4057	229	117	112	0.2
Cargeghe	626	60	30	30	0.52
Castelsardo	5890	413	180	233	0.28
Cheremule	428	24	9	15	0.54
Chiaramonti	1619	102	61	41	0.59
Codrongianos	1311	43	91	-48	0.34
Cossoine	818	41	19	22	0.46
Erula	729	33	10	23	0.56
Esporlatu	382	26	10	16	0.55
Florinas	1509	104	52	52	0.41
Giave	512	16	26	-10	0.67
Golfo Aranci	2471	174	87	87	0.6
Illorai	830	46	25	21	0.37
Ittireddu	483	21	13	8	0.74
Ittiri	8511	633	311	322	0.21
La Maddalena	10961	745	341	404	0.18
Laerru	891	45	19	26	0.63
Loiri Porto San Paolo	3535	285	151	134	0.47
Luogosanto	1839	152	75	77	0.5
Luras	2491	181	95	86	0.55
Mara	586	25	8	17	0.53
Martis	488	22	10	12	0.6
Monteleone Rocca Doria	107	6	3	3	0.66
Monti	2402	200	99	101	0.5
Mores	1912	133	60	73	0.54
Muros	854	79	41	38	0.68
Nughedu San Nicolò	788	42	23	19	0.43
Nule	1365	91	38	53	0.63
Nulvi	2718	197	107	90	0.57
Olbia	59599	5407	2633	2774	0.2
Olmedo	4191	351	160	191	0.39
Oschiri	3225	199	92	107	0.5
Osilo	2951	148	75	73	0.55
Ossi	5724	411	205	206	0.4
Ozieri	10555	734	354	380	0.15
Padria	636	31	16	15	0.52
Padru	2080	149	66	83	0.65
Palau	4135	333	153	180	0.19
Pattada	2999	216	110	106	0.56
Perfugas	2331	160	77	83	0.27
Ploaghe	4464	339	175	164	0.57
Porto Torres	21891	1701	837	864	0.08
Pozzomaggiore	2568	146	73	73	0.19
Putifigari	722	62	32	30	0.61
Romana	538	38	23	15	0.56
San Teodoro	4951	337	164	173	0.66
Santa Maria Coghinas	1370	76	35	41	0.5
Santa Teresa di Gallura	5304	321	152	169	0.16
Sant'Antonio di Gallura	1498	96	44	52	0.44
Sassari	125998	8726	4181	4545	0.14
Sedini	1334	80	36	44	0.39
Semestene	143	4	4	0	0.85

Sennori	7136	521	252	269	0.44
Siligo	843	46	24	22	0.52
Sorso	14517	1114	543	571	0.38
Stintino	1559	84	39	45	0.58
Telti	2295	203	96	107	0.65
Tempio Pausania	13726	916	405	511	0.18
Tergu	608	32	15	17	0.54
Thiesi	2874	201	101	100	0.16
Tissi	2408	213	101	112	0.43
Torralba	946	62	36	26	0.51
Trinitá d'Agultu e Vignola	2315	127	68	59	0.62
Tula	1514	118	54	64	0.74
Uri	2910	209	90	119	0.45
Usini	4314	370	194	176	0.39
Valledoria	4326	278	126	152	0.27
Viddalba	1677	106	50	56	0.54
Villanova Monteleone	2245	148	77	71	0.6
**Total**	**486689**	**35076**	**17028**	**18048**	
